# Prevalence and risk factors for myopia in Taiwanese diabetes mellitus patients: a multicenter case–control study in Taiwan

**DOI:** 10.1038/s41598-021-87499-y

**Published:** 2021-04-14

**Authors:** Hsin-Ting Lin, Cai-Mei Zheng, Yu-Ann Fang, Ju-Chi Liu, Yun-Chun Wu, Yun-Hsiang Chang, Jiann-Torng Chen, Chang-Min Liang, Tian-Jong Chang, Jing-Quan Zheng, Ming-Cheng Tai, Yuh-Feng Lin

**Affiliations:** 1 Department of Ophthalmology, Tri-Service General Hospital, National Defense Medical Center, Taipei, Taiwan; 2 Graduate Institute of Medical Sciences, National Defense Medical Center, Taipei, Taiwan; 3 Graduate Institute of Clinical Medicine, College of Medicine, Taipei Medical University, Taipei, Taiwan; 4 Department of Internal Medicine, School of Medicine, College of Medicine, Taipei Medical University, Taipei, Taiwan; 5 Division of Nephrology, Department of Internal Medicine, Shuang Ho Hospital, Taipei Medical University, Taipei, Taiwan; 6 TMU Research Center of Urology and Kidney, Taipei Medical University, Taipei, Taiwan; 7 Division of Cardiology, Department of Internal Medicine, Shuang Ho Hospital, Taipei Medical University, New Taipei City, Taiwan; 8 Taipei Heart Institute, Taipei Medical University, Taipei, Taiwan; 9 Cardiovascular Research Center, Taipei Medical University Hospital, Taipei, Taiwan; 10 Division of Cardiology, Department of Internal Medicine, School of Medicine, College of Medicine, Taipei Medical University, Taipei, Taiwan; 11 Institute of Epidemiology and Preventive Medicine, College of Public Health, National Taiwan University, Taipei, Taiwan; 12 Graduate Institute of Aerospace and Undersea Medicine, National Defense Medical Center, Taipei, Taiwan; 13 Department of Medicine, Shuang Ho Hospital, Taipei Medical University, Taipei, Taiwan; 14 Department of Critical Care Medicine, Shuang Ho Hospital, Taipei Medical University, Taipei, Taiwan; 15 Department of Internal Medicine, School of Medicine, National Defense Medical Center, Taipei, Taiwan

**Keywords:** Epidemiology, Risk factors, Eye diseases, Diabetes

## Abstract

This population-based retrospective cohort study investigated the prevalence of myopia among patients with Type 1 and Type 2 diabetes mellitus (DM) and evaluate risk factors for myopia in these groups. Records from 2000 to 2012 with at least one year of follow-up from the Taiwan National Health Insurance Research Database were included. This study included 35,538 patients with DM and 71,076 patients without DM. Patients with DM had a significantly higher adjusted hazard ratio for myopia in all age groups and both sexes compared with patients without DM. The subgroup analysis results revealed that the rates of myopia and astigmatism were significantly higher among patients with DM compared with patients without DM aged < 60 years. However, the rates of high myopia or myopia progression to high myopia did not differ significantly between the two groups. These findings indicate that DM is a critical risk factor for myopia and astigmatism among patients aged < 60 years. Therefore, active surveillance and earlier treatment of myopia are critical for patients with DM.

## Introduction

Patients with diabetes mellitus (DM) develop several ocular problems. Common complications include cataracts, diabetic retinopathy, optic neuropathy, and uveitis. DM affects the oculovisual apparatus of the eye and is thus a leading cause of visual loss. Studies have confirmed that fluctuations in refraction occur with changes in blood sugar levels. The Barbados Eye Study^1^ and the Los Angeles Latino Eye Study^2^ have demonstrated that DM is an independent risk factor for myopia^1,2^. A study revealed that poor glycemic control is a major risk factor for myopia^3^. However, a population study reported that the presence of DM was related to an increased shift toward hyperopia^4^.

Patients with DM have a higher surface curvature of the lens compared with individuals without DM. However, the equivalent refractive index of patients with DM is lower; thus, lenticular powers are similar between both patients with DM and those without DM. However, DM was not associated with a shift in ocular refraction in other epidemiological studies (Andhra Pradesh Eye Disease Study [37], Blue Mountains Eye Study [19]). Duke-Elder^5^ suggested that hyperglycemia caused by DM leads to osmotic interactions between the lens materials and the aqueous anterior chamber of the eye^5^. Hyperglycemia affects refraction changes by an osmotic fluid shift, resulting in the hydration of the lens and consequent myopia^6^. Hyperglycemia thus causes myopic refraction, whereas less myopic or hyperopic refraction is associated with low hyperglycemia or hypoglycemia^7–9^. However, other investigators have reported that increased blood glucose resulted in hyperopia instead of myopia^8,10–12^. Hyperopia is common during the earlier phases of hyperglycemia because of a decrease in lens volume, which further proceeds to myopia when lens volume starts to increase^13,14^. An acute or transient rise in serum glucose levels is also related to hyperopia because of changes in the refractive index of the lens^15^. Diverging results have been reported in patients with hyperglycemia and higher HbA1c before intensive sugar control^8,16^.

Findings indicate that myopia is increasingly common, and the global prevalence of myopia is expected to increase from 27% in 2010 to 52% by 2050^17^. Moreover, high myopia (≤ − 6.0 diopters [D]) increases the risk of irreversible vision loss, which is associated with a heavy socioeconomic burden^18^. Clinical studies on the prevalence of refractive errors in diabetic and nondiabetic populations are also controversial. Some studies have revealed no differences in refraction between patients with and without DM^19–21^, whereas another study reported a higher level of myopic refraction in patients with DM^22^. A population-based study conducted in India reported that poor glycemic control was associated with myopia^23^. A regional population study conducted in Taiwan also reported a higher myopia prevalence among patients with DM^24^. However, this study only represented regional data and had a relatively small sample size. Therefore, we aimed to explore risk factors for and the predictors of myopia in the Taiwanese population with DM.

## Materials and methods

### Study cohort

This study was conducted using the largest database of the Taiwanese population, the National Health Insurance Research Database (NHIRD), which covers the health care services of over 99% of Taiwan’s 23 million residents. The NHIRD is currently managed centrally and supervised by the Data Science Center of the Ministry of Health and Welfare. Therefore, the sample tracking time is sufficiently long to perform a complete generational retrospective cohort study. Risk factors for myopia and the relationship between DM and myopia were explored and compared between patients with DM (DM group) and controls (control group). All data in the NHIRD are connected and deidentified. The NHIRD provides information regarding outpatient, inpatient and, emergency visits and drug, and disease diagnosis records of all insured persons and were coded by the International Classification of Disease-9th Revision-Clinical Modification (ICD-9 CM). In addition, the enrollment files of beneficiaries and providers were included. The data period used in this study was 2001 to 2012. The present study had been approved by the Human Research Ethics Committee of Taipei Medical University (TMU-JIRB: N202002017). The Taiwan Ministry of Health and Welfare and Joint Institutional Review Board of Taipei Medical University determined that patient consent was not required because all data were anonymized by the data holder, the Taiwan National Health Insurance Administration (NHIA). Taiwan NHI system, established since 1995, acts as a single-payer insurance system and has been co-funded by Taiwan government, employers, and beneficiaries. Taiwan citizens and stay holders living inside Taiwan for more than 6 months are eligible to be enrolled in NHI. The NHI database is a complete dataset which included comprehensive registration information and claims data since 1995. Patient characteristics, physical examinations, diagnoses, detail information on drugs prescription, surgical operations and procedures, medical costs were recorded in the dataset. Thus, this is the largest national dataset included approximately 23 million beneficiaries with a coverage rate of 99.5% at the end of 2016^25^. Moreover, the whole database was prevented from confidentiality leaks by unique national personal identification which was anonymized and consistent across the NHI database and in between other data sets, allowing valid internal and external linkage^26^. International Classification of Diseases, 9th Revision, Clinical Modification (ICD-9-CM) codes from 1997 through 2015; ICD-10-CM codes since 2016 were used to record the diagnoses and procedures. In our study, all methods were performed as in accordance with the relevant guidelines and regulations approved by the Data Science Center of the Ministry of Health and Welfare and TMU-JIRB.

### Objectives

From January 1, 2001, to December 31, 2012, ICD-9-CM codes (International Classification of Diseases, Ninth Revision, Clinical Modification) were used for outpatient, emergency, and inpatient diagnoses, and patients with DM were thus identified. Criteria for inclusion were as follows: (1) Patients with new onset DM since January 1, 2001 (2) patients with two outpatient diagnoses of DM or one inpatient diagnosis of DM within 1 year after the initial diagnosis and (3) administration of two or fewer oral glycemic drug prescription within 90 days after the initial diagnosis. After selecting patients with DM for inclusion in the study, one comparative group without a diagnosis of DM were randomly selected using age and sex pairing (at a ratio of 1:2). The start date (index date) of the DM group entering the study was set as the time of the first diagnosis of DM, and the same date was set for the matched comparative population. DM group and non-DM control group with following events were excluded (1) those with myopia before the index date and (2) those with cataracts before the index date. After pairing was completed and myopia and cataract before index date were excluded from both study groups, 35,538 patients with DM and 71,076 non-DM population were finally selected.

We included a total of 2174 patients with Type 1 DM, 33,364 patients with Type 2 DM.

### Definitions and variables

The purpose of this study was to investigate the relationship between DM and myopia. Three dependent terms were employed: (1) general myopia (ICD-9-CM code 367.1); (2) progressive high (degenerative) myopia (ICD-9-CM code 360.21); and (3) general myopia, high myopia, or astigmatism (regular astigmatisms with (ICD-9-CM) codes 367.1, 360.21, and 367.21). The start date (index date) was defined as the date when each patient with DM was first diagnosed as having DM during the 2001–2012 study period, whereas the comparison group without DM during the study period was assigned the same start date as that of matched patients with DM. Age and sex were matched at a 1:2 ratio. Among DM and non-DM group who developed general myopia, high myopia, or astigmatism twice within one year, the end time was defined as the date of diagnosis. The end date for other populations was defined as death, loss to follow-up, or the end of the study (December 31, 2012). The tracking time was calculated by subtracting the end time from the index date.

Common variables in this study were demographic characteristics namely age (divided into eight groups: 0–9, 10–19, 20–29, 30–39, 40–49, 50–59, 60–69, and ≥ 70 years), sex (male or female), degree of urbanization (urban, suburban, and rural areas), monthly income (0, 1–21,000, 21,001–33,300, and ≥ 33,301 NTD), and residential area (north, middle, south, east, and outlying islands). The following comorbidities were evaluated using Charlson’s comorbidity index (CCI, 0, 1, 2, and ≥ 3): heart failure (HF; ICD-9-CM code 428), acute myocardial infarction (AMI; ICD-9-CM code 410), stroke (ICD-9-CM codes 430–438), ischemic heart disease (ICD-9-CM codes 410–414), angina (ICD-9-CM code 413), peripheral vascular disease (ICD-9-CM codes 440–448), hypertension (ICD-9-CM codes 401–405), hyperlipidemia (ICD-9-CM code 272), renal failure (ICD-9-CM codes 582, 583, 585, 586, and 588), chronic liver disease (ICD-9-CM codes 456, 571, and 572), chronic obstructive pulmonary disease (ICD-9-CM codes 491, 492, and 496), and cataract (ICD-9-CM code 366).

### Statistical analysis

The significance level (α level) of this study was set at 0.05, and the statistical software packages SAS 9.4 and R (version 3.4.3 × 64) were used for data collation and statistical analysis. Nationwide data were used in this retrospective generational study to explore the relationship between DM and myopia. First, descriptive statistics were used to describe the distribution of demographic variables and comorbidities. The distributions of demographic variables of the DM and control groups are described using statistical values, such as the frequency, percentage, average, and standard deviation. Continuous data are presented as means and standard deviations; categorical data are presented as numbers and percentages. The chi-square test and t test were used to examine whether the DM and control groups populations displayed significantly different demographic distributions. Kaplan–Meier analysis combined with log-rank testing was performed to explore differences in the myopia-free survival rate between patients with Type 1 DM and those with Type 2 DM in the DM group. Cox regression analysis (Cox proportional hazard model) was performed to estimate the difference in the subsequent myopia risk between the DM and control groups. The end of follow-up was defined as the occurrence of myopia, death, loss of follow-up or at the end of the study (December 31, 2012) if no event occurred. The following control variables were used to adjust the model: age, sex, socioeconomic status, comorbidities, and history of living areas. Risk-adjusted hazard ratios and 95% confidence intervals (CIs) were calculated to explore the relationship between DM and myopia and to analyze differences between different types of DM and comparative ethnic groups.

### Ethics statement

We presented a nationwide cohort study by retrieving all patients with diabetes and age, sex matched controls, following up until the development of myopia from January, 2001 to December, 2012, from Taiwan's National Health Insurance Research Database (NHIRD). The NHIRD has been described in detail in previous studies. In brief, it consists of detailed health care data from 23 million enrollees, representing 99% of Taiwan's entire population. For the purpose of protecting patient privacy, our data sources had been ID delinked. In addition, this study had been approved by the ethical review board of the Taipei Medical University, Taiwan (certificate no. TMU-JIRB N202002017).

## Results

### Demographic and clinical characteristics of study participants

**Figure 1:**
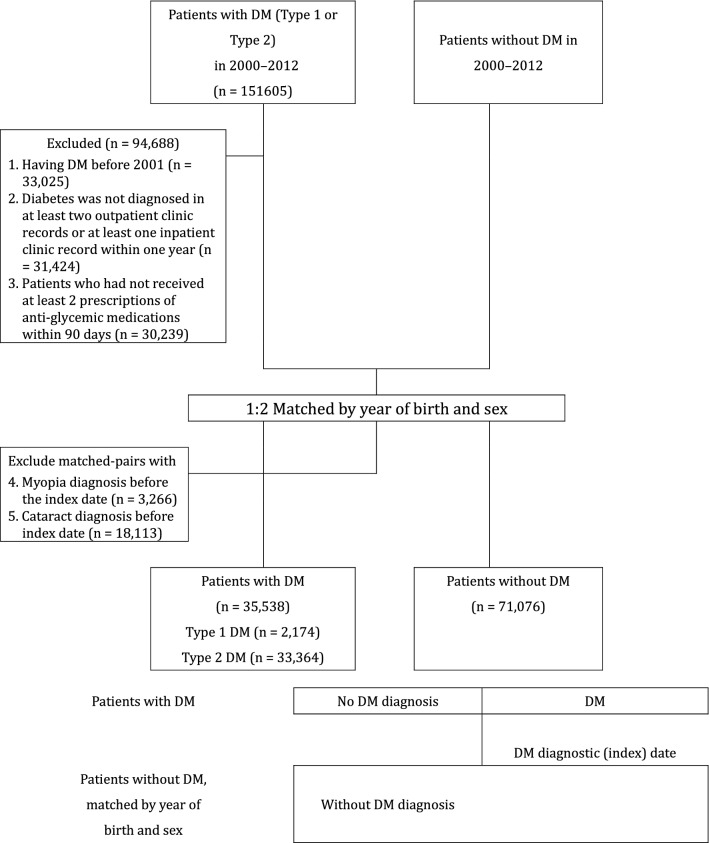
Data selection process. Follow-up of Myopia new-onset; patients with DM were matched with DM-free counterparts, who had the same index dates as the DM diagnostic date.

The flowchart of study participant enrollment is presented in Fig. 1. A total of 151,605 patients were newly diagnosed as having Type 1 or Type 2 DM from 2000 to 2012. Exclusion criteria include: (1) patients diagnosed as having DM before 2001 (n = 33,025), (2) Diabetes was not diagnosed in at least two outpatient clinic records or at least one inpatient clinic record after the first new onset DM within one year (n = 31,424). (3) Patients who had not received at least 2 times prescriptions of anti-glycemic medications within 90 days after new onset of DM (n = 30,239). We therefore randomly recruited 1:2 age and sex matched DM and non-DM pairing and use the new onset DM date as the index date. We then further exclude the following subjects from both DM and non-DM group. (4) Myopia diagnosis before the index date (n = 3266) (5) Cataract diagnosis before index date (n = 18,113). Eligible patients with Type 1 DM (n = 2714) and Type 2 DM (n = 33,364) were finally recruited as the study cohort. The control group include subjects without DM (n = 71,076) was age- and sex-matched with the study group at a 1:2 ratio (Fig. 1). The characteristics and demographic variables of study patients are listed in Table 1. The DM group had significantly higher CCI scores than did the control group (≥ 4 points, 20.08% vs. 15.06%). The DM group had significantly higher rates of comorbid conditions than did the control group, including HF (6.78% vs. 3.99%), AMI (1.34% vs. 0.85%), stroke (13.45% vs. 9.53%), ischemic heart disease (23.32% vs. 15.91%), angina (8.60% vs. 5.84%), peripheral vascular disease (6.96% vs. 5.10%), hypertension (51.29% vs. 30.24%), hyperlipidemia (30.38% vs. 17.74%), renal failure (9.11% vs. 6.53%), chronic liver disease (27.70% vs. 18.56%), and COPD (20.83% vs. 17.58%). Significantly more patients with DM lived in rural (7.75% vs. 7.28%) and suburban areas (17.82% vs. 17.48%). Furthermore, patients with DM had a lower total income (≥ 33,301 NTD, 32.21% vs. 36.02%). More cases of new-onset general myopia were noted in the DM group (1.93% vs. 1.34%). However, the rates of new-onset high myopia or myopia progression to high myopia cases were non-significantly higher in the DM group than in the control group.

**Table 1:** Characteristics of the sample population.

	Control (n = 71,076)		DM (n = 35,538)		*P* ^a^
	n	%	n	%	
**Age, years (mean ± SD)**	51.06 ± 11.01		51.07 ± 11.00		0.926
0–9	84	0.12	41	0.12	0.998
10–19	275	0.39	133	0.37	
20–29	1907	2.68	946	2.66	
30–39	7907	11.12	3931	11.06	
40–49	21,672	30.49	10,879	30.61	
50–59	26,274	36.97	13,094	36.85	
60–69	9852	13.86	4967	13.98	
≥ 70	3105	4.37	1547	4.35	
**Sex**					
Female	28,044	39.46	14,022	39.46	1.000
Male	43,032	60.54	21,516	60.54	
**Charlson’s comorbidity index**					
0	40,043	56.34	16,130	45.39	< 0.001
1	16,769	23.59	9346	26.30	
2–3	7840	11.03	5267	14.82	
≥ 4	6424	9.04	4795	13.49	
**Comorbidities**					
HF	1187	1.67	1237	3.48	< 0.001
AMI	316	0.44	325	0.91	< 0.001
Stroke	3482	4.90	2804	7.89	< 0.001
Ischemic heart disease	6684	9.40	5687	16.00	< 0.001
Angina	2469	3.47	2018	5.68	< 0.001
Peripheral vascular disease	2184	3.07	1666	4.69	< 0.001
Hypertension	14,166	19.93	14,902	41.93	< 0.001
Hyperlipidemia	9553	13.44	9543	26.85	< 0.001
Renal failure	2935	4.13	2299	6.47	< 0.001
Chronic liver disease	11,892	16.73	9597	27.00	< 0.001
COPD	8610	12.11	5264	14.81	< 0.001
Cataract	10,260	14.44	9219	25.94	< 0.001
**Level of urbanization**					
Urban	56,117	78.95	27,760	78.11	0.002
Suburban	11,126	15.65	5711	16.07	
Rural	3833	5.39	2067	5.82	
**Monthly income (NT$)**					
0	2761	3.88	1481	4.17	< 0.001
1–21,000	14,455	20.34	8048	22.65	
21,000–33,300	22,092	31.08	11,838	33.31	
≥ 33,301	31,768	44.70	14,171	39.88	
**Residence**					
North	40,662	57.21	19,240	54.14	< 0.001
Central	14,146	19.90	7543	21.23	
South	14,911	20.98	7952	22.38	
East and other	1357	1.91	803	2.26	
**New-onset myopia**					
Low myopia	1189	1.67	898	2.53	< 0.001
High myopia	193	0.27	97	0.27	0.967
Low/high myopia/astigmatism	1419	2.00	1058	2.98	< 0.001

^a^Categorical variables: Chi-squared test; continuous variables: T test.

### Risks of different myopias between the DM and control groups

The results of the analysis of the incidence and the adjusted hazard ratio (aHR) for myopia in the DM and control groups are presented in Table 2. The incidence rates for myopia, high myopia, myopia progression to high myopia, and myopia-related diseases were 315.8, 41.2, 5.5, and 387.2, respectively, per 100,000 people in the DM group and 215.7, 38.6, 4.5, and 269.0, respectively, per 100,000 people in the control group. The risk of myopia remained significantly higher in the DM group than in the control group after adjustment for demographic characteristics, including age, sex, CCI, comorbidities, socioeconomic status, and area of residence (aHR 1.48; 95% CI .36, 1.61). The risk of myopia-related diseases was also significantly higher in the DM group than in the control DM group (aHR 1.44; 95% CI 1.33, 1.55). The risk of high myopia and myopia progression to high myopia was not significantly different between the two groups. We further stratified the groups according to age and sex. Among the population aged 0–39 and 40–59 years, the risk of general myopia and myopia-related diseases was significantly higher in the DM group than in the control DM group; in the age group of 0–39 years, the risk of general myopia (aHR 1.96; 95% CI 1.67, 2.30) and myopia-related diseases (aHR 1.96; 95% CI 1.68, 2.28) was significantly higher in the DM group than in the control group; in the age group of 40–59 years, the risk of general myopia (aHR 1.48; 95% CI 1.32, 1.65) and myopia-related diseases (aHR 1.44; 95% CI 1.30, 1.60) was significantly higher in the DM group than in the control DM group. However, significant differences in the risk of general myopia and myopia-related diseases were not observed in the age group of > 60 years. The risk of high myopia and general myopia progression to high myopia was not significantly different among all the groups. The risk of myopia and myopia-related diseases was the highest in men with and without DM (aHR 1.89; HR 1.81). The risk of general myopia and myopia-related diseases differed between the DM and control groups in both sexes (female general myopia: aHR 1.36, 95% CI 1.20, 1.54; female myopia-related diseases: aHR 1.29, 95% CI 1.16, 1.44; male general myopia: aHR 1.65, 95% CI  1.48, 1.85; male myopia-related diseases: aHR 1.62, 95% CI 1.47, 1.80).

**Table 2:** Risk of varieties of myopia between DM and control cohorts.

All Groups (n = 106,614)	Control (total follow-up = 467,701.7 person-years)				DM (total follow-up = 228,918.7 person-years)				Adjusted HR^†^ (95% CI)
	no. of patients with myopia	incidence rate (per 10^5^ person-years) (95% CI)			No. of patients with myopia	Incidence rate (per 10^5^ person-years) (95% CI)			
**Whole cohort**									
Low myopia	1,189	254.2	(239.8,	268.7)	898	392.3	(366.6,	417.9)	1.65 (1.51, 1.81)***
High myopia	193	41.3	(35.4,	47.1)	97	42.4	(33.9,	50.8)	0.99 (0.76, 1.28)
Low/high myopia/astigmatism	1,419	303.4	(287.6,	319.2)	1,058	462.2	(434.3,	490.0)	1.59 (1.46, 1.73)***
**Age, 0–39**^a^									
General myopia	124	806.5	(664.5,	948.4)	131	1,807.2	(1497.7,	2116.7)	2.47 (1.91, 3.20)***
High myopia	8	52.0	(16.0,	88.1)	7	96.6	(25.0,	168.1)	1.89 (0.62, 5.79)
Low/High myopia/Astigmatism	132	858.5	(712.0,	1005.0)	138	1,903.8	(1586.1,	2221.4)	2.44 (1.90, 3.14)***
**Age, 40–59**^b^									
Low myopia	981	271.5	(254.5,	288.5)	732	412.8	(382.9,	442.7)	1.73 (1.56, 1.91)***
High myopia	159	44.0	(37.2,	50.8)	76	42.9	(33.2,	52.5)	1.01 (0.75, 1.35)
Low/High myopia/Astigmatism	1,153	319.1	(300.7,	337.5)	850	479.3	(447.1,	511.6)	1.68 (1.53, 1.84)***
**Age, ≥ 60**^c^									
Low myopia	84	92.3	(72.6,	112.1)	35	78.9	(52.8,	105.1)	0.84 (0.56, 1.26)
High myopia	26	28.6	(17.6,	39.6)	14	31.6	(15.0,	48.1)	1.01 (0.51, 1.99)
Low/High myopia/Astigmatism	134	147.3	(122.3,	172.2)	70	157.9	(120.9,	194.8)	1.01 (0.75, 1.36)
**Female**^d^									
Low myopia	564	293.0	(268.8,	317.2)	356	375.3	(336.4,	414.3)	1.56 (1.36, 1.80)***
High myopia	97	50.4	(40.4,	60.4)	46	48.5	(34.5,	62.5)	1.09 (0.75, 1.58)
Low/high myopia/astigmatism	681	353.8	(327.2,	380.4)	430	453.4	(410.5,	496.2)	1.52 (1.34, 1.72)***
**Male**^e^									
Low myopia	625	227.1	(209.3,	244.9)	542	404.3	(370.2,	438.3)	1.89 (1.68, 2.14)***
High myopia	96	34.9	(27.9,	41.9)	51	38.0	(27.6,	48.5)	1.00 (0.70, 1.43)
Low/high myopia/astigmatism	738	268.2	(248.8,	287.5)	628	468.4	(431.8,	505.0)	1.81 (1.62, 2.02)***
**Without cataract**^f^									
Low myopia	1,054	278.7	(261.8,	295.5)	808	531.3	(494.7,	568.0)	1.92 (1.75, 2.12)***
High myopia	140	37.0	(30.9,	43.1)	71	46.7	(35.8,	57.5)	1.30 (0.96, 1.75)
Low/high myopia/astigmatism	1,222	323.1	(305.0,	341.2)	923	606.9	(567.8,	646.1)	1.88 (1.72, 2.05)***
**With cataract**^g^									
Low myopia	135	150.9	(125.5,	176.4)	90	117.1	(92.9,	141.3)	0.75 (0.57, 0.99)*
High myopia	53	59.2	(43.3,	75.2)	26	33.8	(20.8,	46.8)	0.56 (0.34, 0.91)*
Low/high myopia/astigmatism	197	220.2	(189.5,	251.0)	135	175.7	(146.0,	205.3)	0.78 (0.62, 0.98)*

*CI* confidence interval, *HR* hazard ratio.

^†^The main model is adjusted for age, sex, CCI, HF, AMI, stroke, ischemic heart disease, angina, peripheral vascular disease, hypertension, hyperlipidemia, renal failure, chronic liver disease, COPD, cataract, level of urbanization, monthly income, and residence.

* < 0.5, *** < 0.001.

^a^Total follow-up of 7248.8 person-years for the DM group and 15,375.7 for the control group.

^b^Total follow-up of 177,324.3 person-years for the DM group and 361,334.2 for the control group.

^c^Total follow-up of 44,345.6 person-years for the DM group and 90,991.9 for the control group.

^d^Total follow-up of 94,846.5 person-years for DM group and 192,485.7 for the control group.

^e^Total follow-up of 134,072.1 person-years for the DM group and 27,5216.0 for the control group.

^f^Total follow-up of 152,074.4 person-years for the DM group and 378,245.0 for the control group.

^g^Total follow-up of 76,844.3 person-years for the DM group and 89,456.7 for the control group.

### Risk of different myopias among Type 1 DM, Type 2 DM, and control groups

Sensitivity analysis was performed using the Cox risk model to explore differences in the risk of myopia, high myopia, and myopia-related diseases among the Type 1 DM, Type 2 DM, and control groups. The results are presented in Table 3. Compared with the control group, both the Type 1 DM (aHR 2.28; 95% CI 1.89, 2.76) and Type 2 DM (aHR 1.45; 95% CI 1.33, 1.58) groups had a significantly higher risk of myopia, especially in the age groups of 0–39 years (aHR 3.65 in Type 1 DM, aHR 1.67 in Type 2 DM) and 40–59 years (aHR 1.37 in Type 1 DM, aHR 1.49 in Type 2 DM) in both sexes. The risk of myopia-related diseases was significantly higher in both Type 1 (aHR 2.07; 95% CI 1.73, 2.47) and Type 2 DM groups (aHR 1.41; 95% CI 1.31, 1.52), especially in the age groups of 0–39 years (aHR 3.52 in Type 1 DM, aHR 1.70 in Type 2 DM) and 40–59 years (aHR 1.38 in Type 1 DM, aHR 1.45 in Type 2 DM) in both sexes. The results of the subgroup analysis revealed that Type 1 DM was associated with a higher risk of myopia and myopia-related diseases than Type 2 DM in the age group of ≤ 40 years (aHR 3.65 in Type 1 DM vs. aHR 1.67 in Type 2 DM for myopia; aHR 3.52 in Type 1 DM vs. aHR 1.70 in Type 2 DM for myopia-related diseases). Similarly, a higher risk of myopia and myopia-related diseases was observed in patients with Type 1 DM than in patients with Type 2 DM in both sexes (female sex, HR 2.11 in Type 1 DM vs. aHR 1.23 in Type 2 DM; male sex, HR 2.07 in Type 1 DM vs. aHR 1.59 in Type 2 DM). The rates of high myopia and myopia progression to high myopia did not differ significantly between the two types of DM and the control groups in all age groups and both sexes. No significant differences in the risk of myopia, myopia progression to high myopia, and myopia-related diseases were observed among Type 1 DM, Type 2 DM, and control groups in older patients aged ≥ 60 years.

**Table 3:** Risk of various myopia among different types of DM and the control cohort.

	Control (n = 71,076)	Type 1 DM (n = 2,174)	Type 2 DM (n = 33,364)
	Adjusted HR (95% CI)	Adjusted HR (95% CI)	Adjusted HR (95% CI)
**Low myopia**			
Unadjusted	1.00	2.70 (2.22, 3.28)***	1.45 (1.32, 1.58)***
Main model^†^	1.00	2.73 (2.24, 3.32)***	1.66 (1.51, 1.82)***
**Subgroup effects**			
Age, years			
0–39	1.00	4.11 (2.96, 5.71)***	1.81 (1.33, 2.47)***
40–59	1.00	1.88 (1.41, 2.50)***	1.72 (1.55, 1.91)***
≥ 60	1.00	0.71 (0.18, 2.92)	0.85 (0.56, 1.29)
Sex			
Female	1.00	2.95 (2.21, 3.93)***	1.44 (1.24, 1.67)***
Male	1.00	2.55 (1.94, 3.35)***	1.83 (1.62, 2.07)***
Cataract			
Without	1.00	3.07 (2.49, 3.78)***	1.82 (1.65, 2.01)***
With	1.00	1.12 (0.63, 2.00)	0.71 (0.53, 0.95)*
**High myopia**			
Unadjusted	1.00	0.87 (0.39, 1.97)	1.04 (0.81, 1.33)
Main model^†^	1.00	0.83 (0.37, 1.88)	1.05 (0.81, 1.37)
**Subgroup effects**			
Age, years			
0–39	1.00	–	–
40–59	1.00	0.94 (0.38, 2.29)	1.01 (0.75, 1.36)
≥ 60	1.00	0.90 (0.12, 6.71)	1.02 (0.51, 2.04)
Sex			
Female	1.00	0.93 (0.29, 2.97)	1.10 (0.75, 1.61)
Male	1.00	0.75 (0.24, 2.38)	1.02 (0.71, 1.47)
Cataract			
Without	1.00	1.10 (0.41, 3.00)	1.31 (0.97, 1.78)
With	1.00	0.42 (0.10, 1.78)	0.57 (0.35, 0.94)*
Low/high myopia/astigmatism			
Un-adjust	1.00	2.47 (2.06, 2.97)***	1.45 (1.33, 1.57)***
Main model^†^	1.00	2.49 (2.06, 3.00)***	1.60 (1.47, 1.75)***
**Subgroup effects**			
Age, years			
0–39	1.00	3.94 (2.85, 5.44)***	1.84 (1.36, 2.48)***
40–59	1.00	1.80 (1.38, 2.35)***	1.67 (1.52, 1.83)***
≥ 60	1.00	0.85 (0.31, 2.30)	1.02 (0.75, 1.38)
Sex			
Female	1.00	2.69 (2.05, 3.53)***	1.42 (1.24, 1.62)***
Male	1.00	2.33 (1.80, 3.03)***	1.76 (1.57, 1.97)***
Cataract			
Without	1.00	2.94 (2.41, 3.60)***	1.79 (1.63, 1.96)***
With	1.00	0.92 (0.54, 1.56)	0.76 (0.60, 0.97)*

*CI* confidence interval, *HR* hazard ratio.

* < 0.5, *** < 0.001.

^†^The main model is adjusted for age, sex, CCI, HF, AMI, stroke, ischemic heart disease, angina, peripheral vascular disease, hypertension, hyperlipidemia, renal failure, chronic liver disease, COPD, cataract, level of urbanization, monthly income, and residence.

### Predictors of new-onset myopia between DM and control groups

Table 4 lists the findings of univariate and multivariate analyses of variables related to new-onset myopia in the DM and control groups. Age was a significant factor affecting myopia. The risk of myopia decreased with progressive aging. Men had a lower risk of myopia than did women (aHR 0.77; 95% CI 0.72, 0.82). A higher CCI score was associated with a higher risk of myopia. People living in urbanized areas and people with higher incomes (≥ 33,301 NT$) had a higher risk of myopia. People living in northern and eastern areas displayed a significantly higher risk of myopia than did those living in middle and southern areas.

**Table 4:** Predictors of new-onset low myopia, high myopia, and astigmatism in the DM and control groups.

Exposure variable	Univariate analysis				Multivariate analysis			
	*P* value	HR	95% CI		*P* value	HR	95% CI	
DM (ref = control)	< 0.001	1.518	1.402	1.644	< 0.001	1.593	1.464	1.733
**Age, years (Mean ± SD)**								
0–9	< 0.001	72.184	48.154	108.205	< 0.001	60.353	39.238	92.832
10–19	< 0.001	14.214	9.309	21.704	< 0.001	12.277	7.950	18.958
20–29	< 0.001	7.064	4.935	10.111	< 0.001	5.160	3.545	7.511
30–39	< 0.001	5.340	3.821	7.464	< 0.001	3.853	2.710	5.477
40–49	< 0.001	3.559	2.562	4.945	< 0.001	2.522	1.789	3.557
50–59	< 0.001	2.204	1.583	3.070	0.006	1.613	1.146	2.269
60–69	0.070	1.391	0.974	1.985	0.218	1.253	0.875	1.795
≥ 70		1.000				1.000		
**Sex**								
Female		1.000				1.000		
Male	< 0.001	0.857	0.791	0.927	< 0.001	0.726	0.669	0.789
**Charlson’s Comorbidity Index**								
0		1.000				1.000		
1	0.001	1.183	1.076	1.300	< 0.001	1.216	1.097	1.349
2–3	< 0.001	1.252	1.107	1.416	< 0.001	1.383	1.195	1.600
≥ 4	0.144	1.116	0.963	1.292	0.002	1.361	1.119	1.655
**Comorbidities (ref = non-disease)**								
HF	0.014	0.640	0.449	0.912	0.027	0.661	0.459	0.954
AMI	0.901	1.037	0.588	1.828	0.759	1.096	0.612	1.962
Stroke	0.015	0.779	0.638	0.952	0.345	0.902	0.727	1.118
Ischemic heart disease	0.343	0.938	0.823	1.070	0.133	1.142	0.960	1.359
Angina	0.625	0.948	0.765	1.174	0.923	0.987	0.760	1.282
Peripheral vascular disease	0.393	0.901	0.711	1.143	0.803	0.970	0.760	1.236
Hypertension	0.001	0.846	0.770	0.930	0.482	0.961	0.860	1.074
Hyperlipidemia	0.054	1.109	0.998	1.232	0.706	1.023	0.908	1.152
Renal failure	0.779	1.028	0.849	1.244	0.914	1.012	0.819	1.250
Chronic liver disease	< 0.001	1.272	1.155	1.401	0.627	1.031	0.913	1.163
COPD	0.180	1.084	0.963	1.221	0.003	1.204	1.064	1.362
Cataract	< 0.001	0.507	0.451	0.569	< 0.001	0.694	0.612	0.787
**Level of urbanization**								
Urban		1.000				1.000		
Suburban	< 0.001	0.660	0.583	0.747	0.010	0.838	0.733	0.959
Rural	< 0.001	0.600	0.488	0.737	0.607	0.943	0.753	1.180
**Monthly income (NT$)**								
0		1.000				1.000		
1–21,000	< 0.001	0.565	0.462	0.690	0.033	0.783	0.625	0.981
21,000–33,300	< 0.001	0.609	0.504	0.735	0.084	0.822	0.658	1.027
≥ 33,301	0.450	1.071	0.896	1.282	0.002	1.408	1.132	1.752
**Residence**								
North		1.000				1.000		
Central	< 0.001	0.740	0.665	0.822	0.101	0.906	0.806	1.019
South	< 0.001	0.581	0.519	0.651	< 0.001	0.673	0.599	0.756
East and other	0.070	1.242	0.983	1.571	< 0.001	1.578	1.236	2.014

^†^The main model is adjusted for age, sex, CCI, HF, AMI, stroke, ischemic heart disease, angina, peripheral vascular disease, hypertension, hyperlipidemia, renal failure, chronic liver disease, COPD, cataract, level of urbanization, monthly income, and residence.

### Survival analysis: myopia-free survival rate in DM and control groups

**Figure 2:**
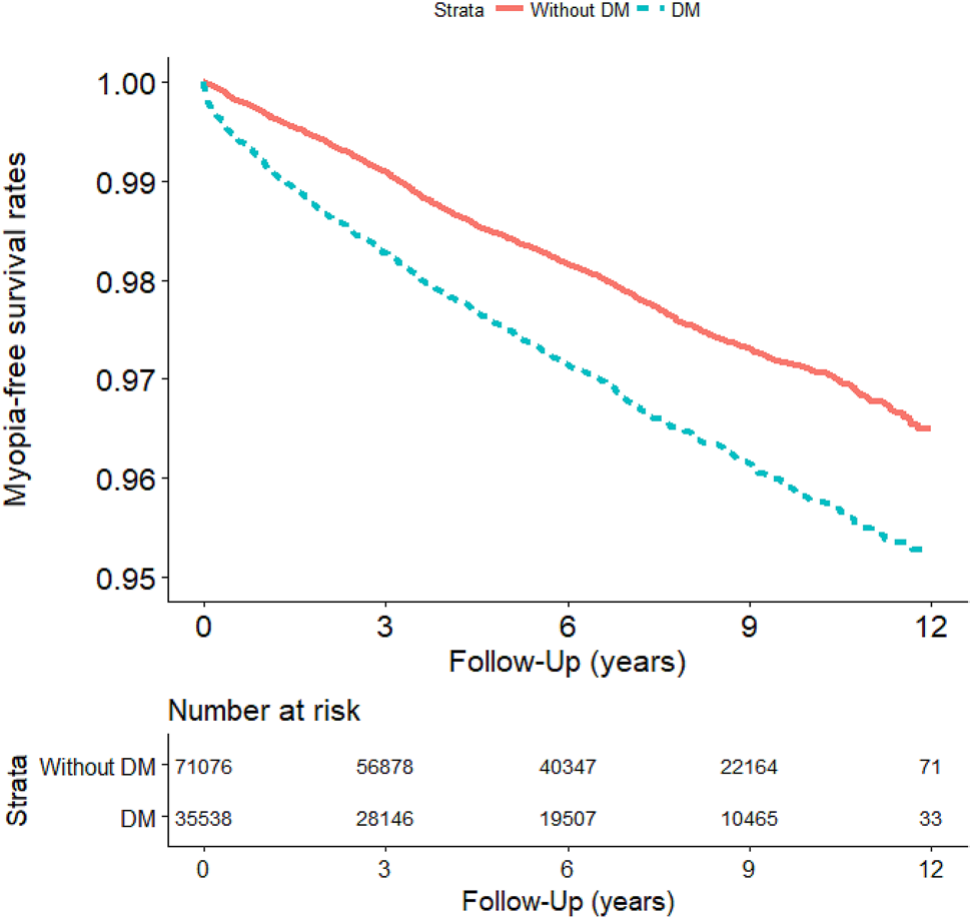
Myopia events in the study cohort (n = 106,614) from January 1, 2001, to December 31, 2012, in Taiwan, stratified by DM and without DM t (log–rank test, χ^2^ = 107.226; df = 1; p < 0.001).

**Figure 3:**
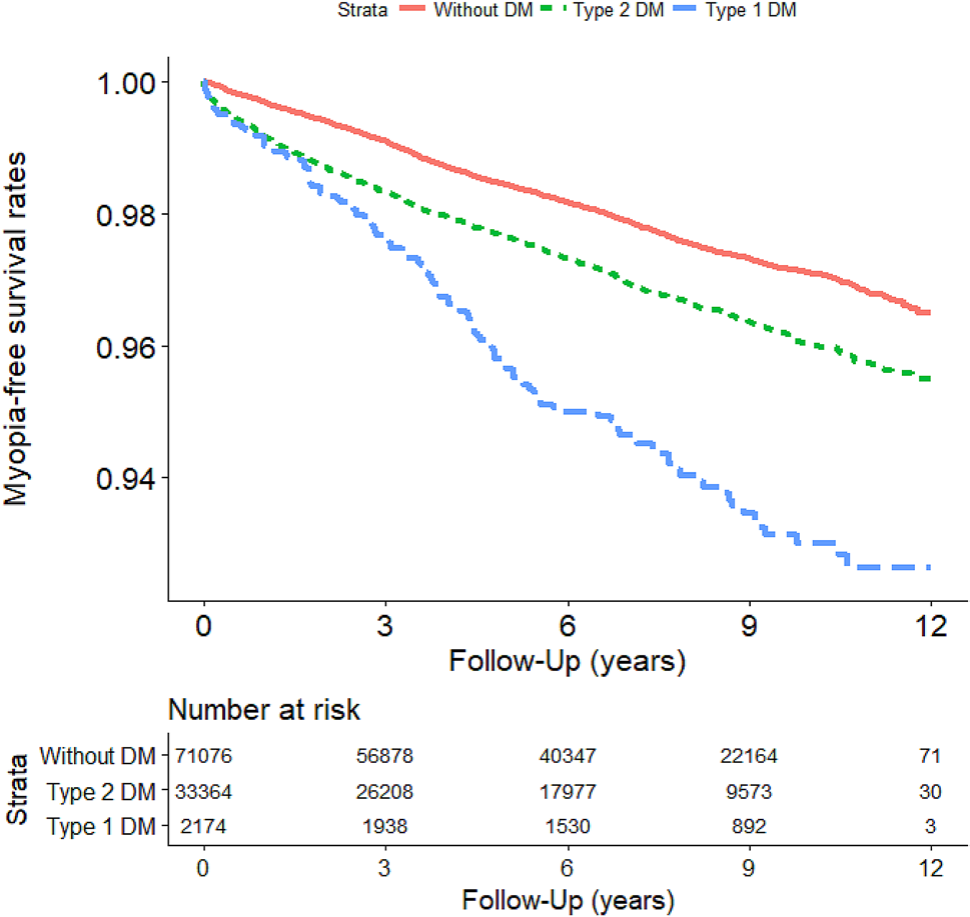
Myopia events in the study cohort (n = 106,614) from January 1, 2001, to December 31, 2012, in Taiwan, stratified by Type 1 DM, Type 2 DM, and without DM (log-rank test, χ^2^ = 148.929; df = 2; p < 0.001).

The difference in the myopia-free survival rate between the DM and control groups is presented in Fig. 2. The survival curve indicated that the rate of myopia in the DM group was significantly higher than that in the control group (*P* < 0.001). The Cox risk model was used to explore differences in the risk of myopia, high myopia, and flash between the DM and control groups. The difference in the myopia-free survival rate between patients with Type 1 DM and Type 2 DM and controls is presented in Fig. 3. Patients with Type 1 DM exhibited the highest myopia-free survival rate, and patients with Type 2 DM displayed a significantly higher rate of myopia than did the control group (*P* < 0.001).

## Discussion

The baseline data of the present study indicated that the DM group had significantly higher Charlson Comorbidity Index scores and more comorbid conditions than did the control group. Compared with controls, significantly more patients with DM lived in rural and suburban areas and they had a significantly lower total income. More cases of new-onset myopia were noted in the DM group than in the control group. However, the numbers of new-onset high myopia and myopia progression to high myopia cases were not significantly higher in the DM group than in the control group. This finding is consistent with that reported by Kinmen who indicated that the prevalence of myopia was higher than that of high myopia or hyperopia.

The adjusted hazard ratios for myopia and myopia-related diseases were significantly higher in the DM group than in the control group (Table 2). Based on the survival curve, we determined that the rate of myopia was significantly higher in the DM group than in the control group (*P* < 0.001; Fig. 2). However, the risk of high myopia and myopia progression to high myopia was not significantly different between the two groups (Table 2). This finding may be because the degree of myopia and the progression of myopia are more highly associated with conditions other than hyperglycemia and hypoglycemia. High myopia is a critical concern because it is associated with scleral thinning^27,28^, which can progress to local outpouchings, staphylomas^29^, and myopic macular degeneration (MMD) if not controlled^30,31^. Staphyloma and MMD represent pathological myopia. Similar findings were noted in both sexes. Furthermore, we analyzed the risk of different myopias in our study cohort. In the population aged 0–39 and 40–59 years, the risk of myopia and myopia-related diseases was significantly higher in the DM group than in the control group (Table 2). However, no such relationship was noted in patients aged ≥ 60 years. Hyperopia is a natural sign of aging. However, myopia is associated with comorbid conditions other than DM status.

We performed a sensitivity analysis by using the Cox risk model to explore differences in the risk of myopia, high myopia, and myopia-related diseases in patients with Type 1 DM, Type 2 DM, and controls (Table 3). The prevalence of myopia in patients with Type 1 DM varied in different populations. Among patients aged ≤ 40 years, Type 1 DM was associated with the highest risk of myopia and myopia-related diseases. Patients with type 2 DM had a higher risk of myopia and myopia-related diseases than did controls. These findings may be caused by myopic errors that evolve from the teenage years in Type 1 DM and can progress up to later years^3,32^. Among patients aged 40–59 years, those with Type 2 DM had significantly higher myopia and astigmatism compared to both patients with Type 1 DM and control. In these patients, the vision status fluctuates with blood sugar levels, and changes in the refractive index of the intraocular lens cause blurred vision^5^. Juvenile cataract is another lens complication related to DM^33^, which is also closely associated with index or transient myopia. Our findings are consistent with those a population study in Danish adults aged 16–66 years; Fledelius^34^ reported that patients with DM had a higher frequency of myopia than did nondiabetic individuals. Another study also demonstrated that myopia and late-onset myopia were more prevalent among patients with DM than among patients without DM^35^. However, in the age group of ≥ 60 years, we determined no significant differences in myopia, high myopia, myopia progression to high myopia, or myopia-related diseases between patients with Type 1 DM and Type 2 DM and controls. This pattern of change indicated that the risk of myopia decreased as an intrinsic age-related decrease in individuals’ myopia, rather than because of diabetic control. Notably, we did not identify significant differences in the rates of high myopia and myopia progression to high myopia between patients with Type 1 DM and those with Type 2 DM and between the DM and control groups in all age and sex groups. Hyperglycemia was not independently related with the degree of myopia or myopia progression, which may be because of genetic and environmental factors.

A comparison of the risk of new-onset myopia between the DM and control groups is presented in Table 4. Age was a significant factor affecting the risk of myopia. The risk of myopia decreased with natural aging among patients with DM. Men also displayed a significantly lower risk of myopia than did women. A higher Charlson Comorbidity Index score was associated with a higher risk of myopia. A higher income and living in the northern and eastern regions of Taiwan or urbanized areas were associated with a higher risk of myopia. Studies have reported that intensive near tasks, downward gaze^36,37^, less time spent outdoors^38^, widespread education, and urbanization are associated with DM. Cataract appears to be protective against onset of myopia, however, it might be possible that some people in the control group had cataract but not had an eye exam. They would then not have been excluded for cataract and seemingly to be protected against myopia.

Our study has limitations. First, a major limitation of the NHIRD is that it does not include laboratory data. Nonetheless, we identified the variables of interest, and the representative population was followed for a long study period with confirmed diagnoses. Second, national databases are reported to have lower accuracy in diagnostic codes compared with clinical charts. Therefore, the National Health Administration cites the standard protocols of diagnosis codes, verifies the accuracy of patients’ diagnoses, and frequently assesses the cross-consistency of claims and chart data. Third, the NHIRD lacks data on lifestyles and habits, including smoking, and is based on the reported data system. Fourth, the results may not represent the entire population because the NHIRD lacks data on the population that is not under medical health care. However, in Taiwan, most health costs are covered by the national insurance, and the missing population is negligible.

To summarize, our population database revealed that DM is an important risk factor for myopia and astigmatism among patients aged less than 60 years. New-onset myopia in a patient with diabetes could be an early indicator of poor DM control and subsequently associated with worsening diabetic retinopathy. Thus, active surveillance and earlier treatment of myopia are critical for patients with DM especially those with poor glycemic control.
